# Puerperal Fever Case

**Published:** 1873-10

**Authors:** J. G. Knox

**Affiliations:** Miss.


### PUERPERAL FEVER CASE.

BY J. G. KNOX, M. D., OF MISS.

In the evening of May 13th, 1873, being called to see Mrs. H., in consultation with Dr. J. H. Blanks, I found her a tolerably heavy set woman, short neck, and of a nervous temperament, age thirty-three, married, had given birth to three living children, and had miscarried once. She had approached the termination of her fifth pregnancy, (counting the miscarriage), and labor had set in about 3 o'clock, P. M., the first intimation of which was a violent convulsion, and an intense pain in the head, which did not fully yield to all the remedies we could bring to bear on it, though it was partially relieved. At first she seemed to be somewhat rational between the convulsions, which occurred perhaps as often as every hour, but her mind seemed to be dull, was very slow in answering questions, frequently they had to be repeated in order to obtain an answer to them. As the pains grew harder, she raved like a maniac in the intervals between the convulsions, every time the pains returned, which was the only rational sign that she was having labor pains, for she was insensible to what was taking place, and to every thing around her. At every return of the pains she would scream and make powerful exertions to get away, so that she had to be held on the bed by main strength. A little over an hour before the child was born, she had such a violent convulsion that it produced a complete dislocation of the inferior maxillary, which was easily reduced in the usual way, after the spasm went off. Dr. Blanks had administered a dose of the bromide of potash soon after she was first taken, and also a dose of oleum ricini. She had had one action on the bowels before I arrived. We immediately took about a quart of blood from the arm, gave her a large dose of magnesia sulph., and waiting awhile we ordered enemata of soapsuds, to be repeated until they produced an action on her bowels, which succeeded only partially, and we failed to procure as free an action as we desired. The pain in the head was still intense, and we applied cut-cups to the temples pretty freely, taking right smart blood. We then gave her tartar emetic in solution, pushing it far enough to produce nausea and slight emesis, and thus thorougly relaxed her system, and under its influence the os uteri and the soft parts dilated rapidly; and when they were nearly dilated, we administered chloroform by inhalation, in order to prevent her struggling and throwing herself about during the pains when the head should enter the pelvis. She was placed on her left side; her husband, who was a strong man, held her firmly in his arms around her waist and shoulders; a pillow was placed between her knees, which were also firmly supported by an assistant. It was a virtex presentation in the first position, (according to Meigs), the pelvis well formed and sufficiently roomy, so that the labor was rapid and easy. We had a dose of the wine of ergot ready, which was given to her as soon as the child was born, to guard against hemorrhage, and also to secure a speedy delivery of the placenta, which came away in a few minutes. The child was born about 11 o'clock at night. She then remained pretty quiet for about an hour, and showed some signs of returning consciousness. The crying of the child seemed to arouse her a little, and she enquired what it was, and when informed, seemed only faintly to realize what had taken place. This calm caused us to indulge the vain hope, that as the exciting cause was removed, that perhaps the convulsions would not return, but we were not permitted to indulge this pleasing hope very long, for they soon returned again, with nearly all of their former violence, though at gradually lengthened intervals, and were followed by a comatose condition, lasting half an hour, sometimes perhaps longer. We watched her all night, giving the bromide of potash in about fifteen grain doses every three hours. When the convulsions returned after the calm spoken of above, we gave her a pretty good dose of morphine, thinking that they might be kept up by after-pains. We would have given her veratrum viridi, to help control the action of the heart, but we did not have it with us. About night she had a very hard one, followed by a deep comatose condition, and we bled her again, taking about a pint of blood this time. I now left her in charge of Dr. Blanks, who agreed to watch her, and have the course of treatment agreed on, carried out, which was to keep her bowels open with some simple purgative, such as oleum ricini, or magnesia sulph. dissolved in a decoction of senna and manna, let her diet be very light for several days, and to control the action of the heart with veratrum viridi, in doses of three or four drops, more or less, as might be necessary to bring down her pulse to about seventy beats per minute, and to keep them at that, until the convulsions and all the cerebral symptoms ceased. She continued to have them at gradually longer intervals till about ten o'clock that night, when they ceased altogether, though she complained of pain, and a full feeling in the head for several days, which gradually gave way. After ten or fifteen days, she was given quinine and iron three times a day to enrich her blood again, and strengthen her. She and the child are both doing well now.

---

Carbolic Acid in Whooping Cough.—Dr. C. Glen Bott (Med. Times and Gazette, June 26, 1872), has found carbolic acid to have wonderful power in arresting whooping-cough. He gives 1-24th to 1-36th of a drop freely diluted with water every four hours to a child eight years old, or in some cases $\frac{1}{8}$ of a drop three times a day to a child four years old.

